# Post-Treatment Follow-Up Study of Abdominal Cystic Echinococcosis in Tibetan Communities of Northwest Sichuan Province, China

**DOI:** 10.1371/journal.pntd.0001364

**Published:** 2011-10-25

**Authors:** Tiaoying Li, Akira Ito, Renqing Pengcuo, Yasuhito Sako, Xingwang Chen, Dongchuan Qiu, Ning Xiao, Philip S. Craig

**Affiliations:** 1 Institute of Parasitic Diseases, Sichuan Centers for Disease Control and Prevention, Chengdu, Sichuan Province, People's Republic of China; 2 Cestode Zoonoses Research Group, School of Environment and Life Sciences, University of Salford, Salford, United Kingdom; 3 Department of Parasitology, Asahikawa Medical University, Asahikawa, Japan; 4 Shiqu County Centers for Disease Control and Prevention, Shiqu, Ganzi Tibetan Prefecture, Sichuan Province, People's Republic of China; Universidad Peruana Cayetano Heredia, Peru

## Abstract

**Background:**

Human cystic echinococcosis (CE), caused by the larval stage of *Echinococcus granulosus*, with the liver as the most frequently affected organ, is known to be highly endemic in Tibetan communities of northwest Sichuan Province. Antiparasitic treatment with albendazole remains the primary choice for the great majority of patients in this resource-poor remote area, though surgery is the most common approach for CE therapy that has the potential to remove cysts and lead to complete cure. The current prospective study aimed to assess the effectiveness of community based use of cyclic albendazole treatment in Tibetan CE cases, and concurrently monitor the changes of serum specific antibody levels during treatment.

**Methodology/Principal Findings:**

Ultrasonography was applied for diagnosis and follow-up of CE cases after cyclic albendazole treatment in Tibetan communities of Sichuan Province during 2006 to 2008, and serum specific IgG antibody levels against *Echinococcus granulosus* recombinant antigen B in ELISA was concurrently monitored in these cases. A total of 196 CE cases were identified by ultrasound, of which 37 (18.9%) showed evidence of spontaneous healing/involution of hepatic cyst(s) with CE4 or CE5 presentations. Of 49 enrolled CE cases for treatment follow-up, 32.7% (16) were considered to be cured based on B-ultrasound after 6 months to 30 months regular albendazole treatment, 49.0% (24) were improved, 14.3% (7) remained unchanged, and 4.1% (2) became aggravated. In general, patients with CE2 type cysts (daughter cysts present) needed a longer treatment course for cure (26.4 months), compared to cases with CE1 (univesicular cysts) (20.4 months) or CE3 type (detached cyst membrane or partial degeneration of daughter cysts) (9 months). In addition, the curative duration was longer in patients with large (>10 cm) cysts (22.3 months), compared to cases with medium (5–10 cm) cysts (17.3 months) or patients with small (<5 cm) cysts (6 months). At diagnosis, seven (53.8%) of 13 cases with CE1 type cysts without any previous intervention showed negative specific IgG antibody response to *E. granulosus* recombinant antigen B (rAgB). However, following 3 months to 18 months albendazole therapy, six of these 7 initially seronegative CE1 cases sero-converted to be specific IgG antibody positive, and concurrently ultrasound scan showed that cysts changed to CE3a from CE1 type in all the six CE cases. Two major profiles of serum specific IgG antibody dynamics during albendazole treatment were apparent in CE cases: (i) presenting as initial elevation followed by subsequent decline, or (ii) a persistent decline. Despite a decline, however, specific antibody levels remained positive in most improved or cured CE cases.

**Conclusions:**

This was the first attempt to follow up community-screened cystic echinococcosis patients after albendazole therapy using ultrasonography and serology in an endemic Tibetan region. Cyclic albendazole treatment proved to be effective in the great majority of CE cases in this resource-poor area, but periodic abdominal ultrasound examination was necessary to guide appropriate treatment. Oral albendazole for over 18 months was more likely to result in CE cure. Poor drug compliance resulted in less good outcomes. Serology with recombinant antigen B could provide additional limited information about the effectiveness of albendazole in CE cases. Post-treatment positive specific IgG antibody seroconversion, in initially seronegative, CE1 patients was considered a good indication for positive therapeutic efficacy of albendazole.

## Author Summary

Cystic echinococcosis is a serious public health problem in Tibetan communities of northwest Sichuan Province, China. Antiparasitic treatment with albendazole remains the only choice in most cases, due to the poor socio-economy and inadequate hospital facilities in this area. A post-treatment follow-up study was carried out in community-detected 49 CE cases by application of abdominal ultrasound and serology with recombinant antigen B (rAgB) in a Tibetan region of Sichuan from 2006 to 2008. Following 6 to 30 months regular albendazole therapy, 32.7% of CE cases were considered cured at ultrasound, 49.0% were classed as improved, 14.3% remained unchanged or static, and 4.1% of cases became aggravated. The treatment course for cure was longer in patients with CE2 type cyst pathology compared to cases with CE1, CE3a or CE3b type cysts. In addition, patients with large cysts (≥10 cm) had a longer curative duration compared to those with medium cysts (5–10 cm) or small cysts (<5c m). The changes of serum specific IgG antibody levels against rAgB were not strongly associated with the viability of cystic echinococcal lesions, however, post-treatment specific IgG antibody positive sero-conversion in initially seronegative CE1 patients, was an indicator for the albendazole efficacy in specific CE patients.

## Introduction

Human cystic echinococcosis (CE), caused by the metacestode stage of *Echinococcus granulosus*, is a complex, chronic disease with a cosmopolitan distribution, and the liver is the most frequently affected organ [1]. Clinical manifestation of this disease ranges from asymptomatic infection to severe, or rarely even fatal disease. Diagnosis of CE remains highly dependent on imaging techniques, due to the fact that immunodiagnosis frequently lacks sensitivity [2], with about 20% of clinically or surgically confirmed CE cases, and up to 50% of community-detected patients presenting negative serology [3]–[6]. The most common applied imaging techiniques include magnetic resonance imaging (MRI), ultrasonography (US) or radiography, for detection of characteristic space-occupying cysts [7], [8]. MRI is able to show highly specific features of CE, but it is prohibitively expensive and not available in rural areas of many endemic countries. In contrast, US is accessible, much less expensive, and can identify hydatid cyst pathological type (CE1-CE5) [9].

Approaches in clinical management for CE include surgery, percutaneous techniques and antiparasitic treatment for active cysts, and the so-called `watch and wai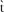 approach for inactive cysts [9]. Currently, surgery remains the most common approach for CE treatment that has the potential to remove cysts and lead to complete cure, but it involves risks including those associated with any surgical intervention, anaphylactic reactions, and secondary CE owing to spillage of viable parasite (protoscoleces) material [10]–[12]. Drug therapy with benzimidazoles (albendazole or mebendazole) has increasingly been used to treat CE, and proved to have efficacy against the parasite in humans, with about 30% of patients cured and 30%–50% of cases improved after 12 months follow-up [10]. However, the response to drug therapy is unpredictable, and the optimum duration has not been definitively determined [11], [13]. Moreover, risk of recurrence remains the major problem in surgical or medical treatment [10], [12], [13]. Therefore, post-treatment or post-surgical follow-up of CE patients for several years is usually indicated.

Imaging techniques such as MRI, X-ray or ultrasonography, are useful tools for follow-up of CE patients. However, these techniques are sometimes difficult to detect the newly growing small cyst and also to discriminate between dead and viable cysts [14]. Therefore, efforts have been directed at applying immunological tests of significantly diagnostic and prognostic values. ELISA and immunoblotting for serum antibody detection using various antigen preparations, including crude hydatid cyst fluid, purified fractions of antigen 5 or B, and *E. granulosus* protoscolex soluble extract, have been applied to follow up CE patients [15]–[20]. However, all of these tests exhibited problems mainly related to temporally delayed reactions to clinical changes [18], [20]. Recombinant antigen B (rAgB) proved to have similar diagnostic value to native antigen B in CE patients [21], [22]. However, there has been little or no application of rAgB for post-treatment follow-up of CE patients.

In Tibetan regions of China, human cystic echinococcosis is highly endemic [23]. Albendazole therapy is the primary choice of treatment in the majority of patients owing to remote communities, poor socioeconomics and basic hospital facilities in Tibetan Autonomous Prefectures/communities. The current prospective study was designed to assess the effectiveness of cyclic albendazole treatment in community detected CE patients using ultrasonography as well as ELISA with rAgB as diagnostic/follow-up tools, and also to monitor the changes of serum specific IgG antibody levels against rAgB in these patients during treatment.

## Materials and Methods

### Ethics statement

The study protocol was approved by the Ethical Committee of Sichuan Centers for Disease Control and Prevention (Sichuan CDC). Clearance to carry out the study was obtained from Shiqu County CDC. Information about the purpose of the post-treatment follow-up study was spread to the villagers. Persons with confirmative ultrasound images of CE were voluntarily self-selected to be involved in this study by written informed consent and were assured free medical treatment with cyclic albendazole therapy if necessary. Recommendations were also provided for possible surgical intervention (cyst removal). At confirmed ultrasound diagnosis, each patient was requested to complete a questionnaire which was designed to get information on demographics. Questions were mainly designed to identify clinical manifestations, history of any previous treatment with albendazole (regular or irregular, duration), as well as history of surgery. At each follow-up, another questionnaire was completed to obtained information on administration of albendazole, surgery associated with echinococcosis, improvement of symptoms if any, adverse effects such as gastrointestinal disturbances, alopecia, jaundice, skin itch, hepatic pain/sting, dizziness etc. Chinese-Tibetan translators were employed when necessary.

### Criteria for diagnosis and classification of cystic echinococcosis

**Figure 1:**
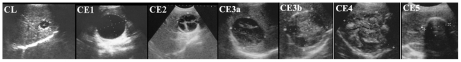
Ultrasound classification of cystic echinococcosis based on WHO expert consensus. CL: as a potentially parasitic cyst, indicates a very early stage of parasite development. CE1 or CE2: suggests active parasite. CE3a: is characterized by detachment cyst membrane and/or partial degeneration of cyst content, without daughter cysts, indicates a transitional stage. CE3b: suggests a transitional stage of parasite, partial degeneration of daughter cysts. CE4 or CE5: indicates an inactive parasite.

Diagnosis and classification of cystic echinococcosis (CE) was made using portable ultrasound according to the criteria proposed by the World Health Organization Informal Working Group on Echinococcosis for CE [12], [24]. On the basis of patho-morphological features of cysts, CE lesions were differentiated into six types: CL, CE1, CE2, CE3, CE4 and CE5 (Figure 1). Briefly, the CL type cyst refers to a cystic lesion of parasite origin without a clear rim indicating a very early stage of parasite development, while the presence of CE1 (unilocular cyst with thick endo membrane) or CE2 (daughter cysts present) is suggestive of active stages of the disease. While CE3 is broken into CE3a and CE3b characterized by detached cyst membrane and partial degeneration of daughter cysts, respectively, indicating the parasite is at a transitional stage, and CE4 and CE5 implies cyst involution, necrosis, partially calcified or inactive parasite [1], [12], [24].

Application of chest X-ray for diagnosis of lung CE was not carried out in this study.

### Origin of cystic echinococcosis (CE) patients

During May 2006 to November 2008, mass ultrasound examination was carried out in eight Tibetan townships of Shiqu County (Ganzi Prefecture, Sichuan Province) for detection of individuals with abdominal cystic echinococcosis infection. Patients with CE1/CE2/CE3a/CE3b type cysts were invited to enroll in the current prospective follow-up study. All CE patients, whether enrolled or not, were offered free albendazole treatment.

### Albendazole therapy

Cyclic treatment with albendazole was provided freely to each patient as 100-mg tablet at a daily dose of 10–15 mg/kg body weight (in two divided doses, together with fat-rich meal). Cyclic treatment of 30 days was followed by a ‘wash out’ period of 7–10 days without albendazole [1]. Albendazole tablets sufficient for six months application were delivered to patients at each follow-up, to whom possible adverse effects were explained. In addition, albendazole was also available freely in the local county CDC clinic.

Follow-up was carried out at six months intervals. Once a cystic lesion changed to CE4 type, the patient was requested to cease albendazole, but further regular ultrasound examination was necessary to understand if the cyst remained inactive. According to the questionnaire investigation, patients who took albendazole as requested during follow-up period were included in the regular-treated group, whereas others who did not take albendazole as requested due to poor compliance belonged to the irregular-treated group.

### Responses to albendazole therapy

The effectiveness of albendazole in CE patients assessed by ultrasound was described as follows: cured, improved, unchanged/static or aggravated. “Cured” was defined as disappearance of cysts, or degeneration of cyst contents. In other words, ‘cured’ referred to a cyst changing to a CE4 or CE5 type from a CE1, CE2, CE3a or CE3b type cyst. ‘Improved’ was determined as detachment of cyst membrane, partial degeneration of cyst contents (or daughter cysts) and/or reduction of cyst size, indicative of the cyst converting to CE3a/CE3b type from a CE1 or CE2 cyst. A ‘static’ or unchanged cyst showed no morphological and/or size changes. ‘Aggravated’ CE disease was defined as enlargement of the cyst and/or recurrence of daughter cysts.

### Collection of serum samples and image data

Approximately 3 ml of venous blood was taken voluntarily from patients at diagnosis (during mass ultrasound screening) as well as at each follow-up, and then centrifuged on the same day. Sera were aliquoted and stored at −20°C for later serological analysis. Blood transaminase levels were not monitored in the current study, due to the difficulty of doing liver function tests in the field.

Information about the characteristics of hydatid cysts for new CE cases and follow-up CE patients was documented in detail, including the cyst type (CE1-5), the number of cysts (single or multiple), location (the lobe of the liver, abdominal cavity, pelvic cavity, spleen or kidney), and the size (cm).

### Serology

ELISA with recombinant antigen B (rAgB) based on previous description [22] was performed on each serum sample for determination of *Echinococcus* specific IgG. Samples from the same patient were analyzed concurrently. The cut-off point was determined as the mean optical density plus 3 times standard deviation for a panel of serum samples obtained from healthy donors (n = 30).

In these assays, 100-µl volume was applied throughout unless otherwise stated. 96-well microtiter plates (MaxiSorp; Nalge Nunc International, Roskilde, Denmark) were coated with diluted rAgB at 0.5 µg/ml in PBS overnight at 4°C. Plates were rinsed 3 times with PBST and blocked with 300 µl of 1% casein buffer at 37°C for 1 hr. Sera were diluted 1∶100 in 1% casein buffer. Plates with diluted sera were incubated in duplicate wells at 37°C for 1 hr and then washed five times with PBST. Rabbit anti-human horseradish peroxidase-conjugated protein G (Zymed Laboratories, Inc., South San Francisco, Calif.) was diluted at 1∶4000 in 1% casein buffer and incubated at 37°C for 1 hour. Plates were washed five times with PBST. For colour development, substrate solution (0.4 mM 2,2′-azino-bis[3-ethybenzthiazoline-6-sulfonic acid] in 0.1 M citric acid buffer and 0.2 M Na_2_HPO_4_) was added into each well and incubated at room temperature for 30 min. Colour reaction was then stopped by application of 1% SDS in each well. The optical density at 405 nm was evaluated with an ELISA reader.

### Statistical analysis

Chi-square test was used to compare the occurrence rate of spontaneous involution between males and females, and the cure rate between the patient groups with albendazole course ≤6 months and those >6 months, ≤12 months and >12 months, ≤18 months and >18 months, and ≤24 months and >24 months. Significance was set at *P*≤0.05.

## Results

A total of 196 persons with CE infection were registered in this study (male = 83, female = 113), with a mean age of 37.5 years at diagnosis (range 4–80 years). Of these 196 cases, 55 (28.1%) had received prior regular albendazole therapy, while an additional 15 (8.2%) had prior surgery. However, only 4 of 15 operated cases received regular albendazole treatment following surgery.

Totally 98 of 108 CE patients without any previous albendazole treatment at diagnosis were investigated about clinical symptoms and signs, 55.1% (54) reported various degrees of discomfort, while 44.9% (44) were asymptomatic. The most common discomfort was hepatic or epigastric pain in 49.0% of patients, other complaints included abdominal distention, palpable abdominal mass, etc. All the 32 patients with cysts of CL, CE4 or CE5 type were asymptomatic, while 74.2% (49/66) of patients with cysts of CE1, CE2 or CE3 presented various degrees of symptoms.

Of these 196 cases, 37 (18.9%) were observed at first examination, to have evidence of spontaneous involution of cystic lesions without any interventional procedures, presenting inactive cysts (CE4 or CE5) in the liver. Persons with CE4 cysts had a mean age of 39.2 years (n = 27), while individuals with CE5 cysts had an average age of 61.3 years (n = 10). Of the 37 patients with evidence of spontaneous involution, 23 were male and 14 were female. In other words, spontaneous cure of cystic echinococcosis occurred more frequently in male (27.7%) than in female (12.4%), and the difference was significant (χ^2^ = 7.3, *P*<0.01).

A total of 49 CE patients received regular albendazole treatment for 6 to 30 months, including 19 males and 30 females. The youngest CE case was 4 years old and the oldest was 80 years, with a mean age of 37.7 years. Cystic lesions were confined in the liver in 43 cases, and the remaining 6 cases had lesions not only to the liver, but also in the abdominal cavity. Of these 49 patients, 16 had CE1 type cysts, 17 had CE2 cysts, 10 had CE3a type cysts, and the remaining 6 had CE3b cysts (Table 1). The cyst measured ≥10 cm in 25 patients, the cyst varied in size 5 cm–10 cm in 20 cases, whereas the remaining 4 CE cases had cysts less than 5 cm (Table 1).

**Table 1:** The cyst stage and size in 49 CE cases.

Size	No. cases				Total
	CE1	CE2	CE3a	CE3b	
Small	2	1	1	0	4
Medium	8	4	6	2	20
Large	6	12	3	4	25
Total	16	17	10	6	49

Small: the cyst measured <5 cm at maximum diameter;

Medium: the cyst measured 5–10 cm at maximum diameter;

Large: the cyst measured ≥10 cm at maximum diameter.

Following 6 to 30 months regular therapy, 16 (32.7%) of 49 patients were observed to have cysts that changed to CE4 type (ie. considered cured), 24 (49.0%) were observed to have cysts converted to CE3a or CE3b from CE1 or CE2 type (ie. improved) (mean duration = 14 months), cysts remained unchanged in the other 7 (14.3%) patients (mean = 10.3 months), and enlargement of hydatid cysts was observed in the remaining 2 (4.1%) patients (Figure 2.1; Table 2). The cure rate was 15.4% (2/13) and 38.9% (14/36) in the patient groups with albendazole course ≤6 months and those >6 months, 21.4% (6/28) and 47.6% (10/21)) for the group with treatment duration ≤12 months and those >12 months, 22.9% (8/35) and 51.7% (8/14) for cases with treatment course ≤18 months and those >18 months, and 26.8% (11/41) and 62.5% (5/8) for patients with albendazole course ≤24 months and those >24 months. Further statistical analysis revealed that the cure rate was significantly different only between the patient group with treatment course ≤18 months and those >18 months (χ^2^ = 5.24, *P*<0.05). The 16 ‘cured’ patients were composed of 5 CE1 cases, 5 CE2, 4 CE3a and 2 cases with CE3b cyst. The treatment course for cure varied in patients with cysts at different stages, that is, the mean curative course was 26.4 months in CE2 patients, 20.4 months in CE1 cases and 9 months in CE3a/CE3b patients. Moreover, the curative duration also differed in cases with cysts at different size. Patients with large hydatid cysts (≥10 cm) needed 22.3 months treatment (n = 7), whereas cure was achieved following 17.3 months therapy in cases with medium cysts (5 cm to 10 cm) (n = 8) and 6 months in patients with small cysts (≤5 cm) (n = 1). In addition, 5 of these 16 cured CE patients were further followed up with ultrasound for 6 to 24 months, in whom the cysts remained inactive (CE4 type), indicative of no recurrence.

**Figure 2:**
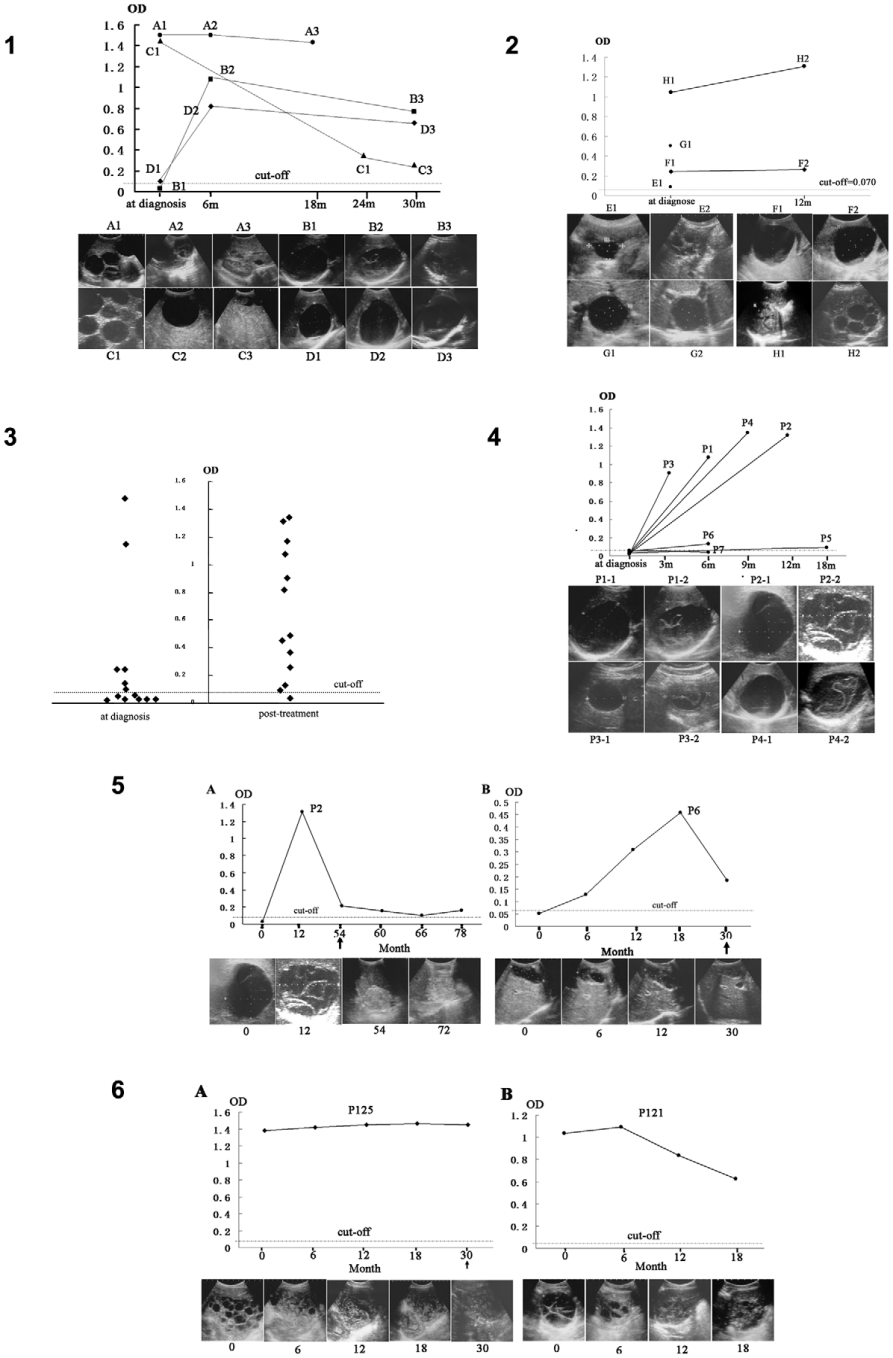
Changes of images and serum antibody levels against rAgB in CE cases during albendazole therapy. **1**. Patients with regular treatment. (A): Hepatic CE2 was cured after 18 months treatment (A-1: at diagnosis; A-2: 12 months; A-3: 18 months). (B): Hepatic CE1 was cured following 30 months treatment (B-1: at diagnosis; B-2: 6 months; B-3: 30 months). (C): CE2 was improved in abdominal cavity following 42 months treatment (C-1: at diagnosis; C-2: 36 months; C-3: 42 months). (D): Hepatic CE1 was aggravated following 30 months treatment (D-1: at diagnosis; D-2: 6 months; D-3: 30 months). **2**. Patients with poor compliance of treatment. (E): Hepatic CE (E-1: CL type cyst at diagnosis; E-2: CE3 type cyst with daughter cyst at 30 months). (F): Hepatic CE (F-1: CE1 type cyst at diagnosis; F-2: unchanged cyst at 12 months). (G): Hepatic CE (G-1: CE1 type cyst at diagnosis; G-2: the unchanged cyst at 6 months). (H): Hepatic CE (H-1: CE3 type cyst at diagnosis; H-2: CE2 type cyst at 12 month). Patient E and G refused to donate blood samples when followed up. **3**. ELISA OD values in 13 CE1 cases before and after treatment (3 months to 18 months). **4**. In seven CE1 cases (P1-P7) with seronegative response at diagnosis, six (P1-P6) converted to be seropositive following 3 months to 18 months treatment. Concurrently the cyst changed to CE3a from CE1 type in all these six cases. **5**. In two albendazole-cured CE1 cases, specific antibody levels were initially elevated and subsequently declined during treatment, but remained positive when CE was cure. **6**. In two albendazole-cured or improved CE2 patients, (A): stability of specific antibody levels in a cured CE2 case; (B): initial elevation followed by subsequent decline of specific antibody levels in an improved CE2 patient. The arrow referred to the time when CE was found to be cured.

**Table 2:** Outcomes of cyclic albendazole treatment in 49 CE cases.

Course (month)	No. cases treated	Cured		Improved		Static		Aggravated	
		No. cases	%	No. cases	%	No. cases	%	No. cases	%
6	13	2	15.4	7	53.8	3	23.1	1	7.7
12	15	4	26.7	8	53.3	3	20.0	0	0
18	7	2	2/7	4	4/7	1	1/7	0	0
24	6	3	3/6	3	3/6	0	0	0	0
30	8	5	5/8	2	2/8	0	0	1	1/8
Total	49	16	32.7	24	49.0	7	14.3	2	4.1

In contrast, 12 CE patients who poorly complied with albendazole treatment were observed to have much poorer prognosis during 6 to 30 months follow-up observation (mean  = 17.0 months). Of these 12 patients, cysts remained unchanged in 8 cases, while enlargement of the cyst or recurrence of daughter cysts was observed in the remaining 4 patients (Figure 2.2).

Of 13 CE1 patients without any previous albendazole treatment, 7 (53.8%) were sero- negative for a specific IgG response to rAgB (Figure 2.3). However, following 3 to 18 months albendzole therapy, positive IgG seroconversion was observed in 6 (no. p1-p6) of these 7 initially seronegative cases (Figure 2.3 and figure 2.4). Concurrently, ultrasound scan detected detachment of cyst membrane and/or partial degeneration of cyst content (i.e. CE1 type changed to CE3a type) in all these six patients (no. p1-p6) (Figure 2.4). In another patient (no. p7) in whom serum specific IgG antibody remained negative during 6 months follow-up period (Figure 2.4), ultrasound scan did not detect any changes of the cyst (the image was not shown). A questionnaire investigation revealed that this patient had reported a poor compliance with albendazole therapy.

Sequential serum samples (n = 36) were obtained from 8 CE patients (CE1 = 4, CE2 = 3 and CE3b = 1), who did not receive any previous chemotherapy at diagnosis and were considered to be cured according to ultrasound images following albendazole therapy in the current study. At least 3 serum samples were monitored in each patient. The follow-up period ranged from 24 months to 78 months (mean 39.8 months), and an average 4.5 serum samples were taken from each patient. Longitudinal assessment of specific IgG antibody against rAgB in ELISA revealed that serum antibody levels of IgG were initially elevated, and subsequently decreased in five (4 CE1 and 1 CE2) of these 8 patients (Figure 2.5). In another two patients (1 CE2 and 1 CE3b), specific IgG antibody levels decreased but with a minor fluctuation during albendazole administration. In the remaining patient with a CE2 type cyst, a cured CE end-point was achieved following treatment, but there was no significant change of specific IgG antibody levels during the period of treatment (Figure 2.6A). Serum specific IgG antibody in fact remained positive in all the 8 patients where CE was considered ‘cured’ even 24 months after cure in one CE patient (ie. P2) (Figure 2.5A).

Consecutively collected 25 sera from 6 patients (CE1 = 2 and CE2 = 4) with improved CE following albendazole treatment were also assessed for specific IgG antibody against rAgB. Of these 6 cases, four (1 CE1 and 3 CE2) did not receive any previous albendaozle therapy at diagnosis, and initial elevation and subsequent decline of specific IgG antibody levels occurred in all these 4 patients (Figure 2.6B). For the other 2 cases with previous albendazole treatment at diagnosis, all antibody levels were observed to progressively decrease following therapy. In contrast, specific IgG antibody levels were observed to sharply increase in one patient with evidently aggravated CE following irregular albendazole administration, presenting enlargement of the cyst and recurrence of daughter cysts (Figure 2.2 H).

Of 83 CE patients who were investigated about adverse effects related to albendazole administration, 37 (44.6%) reported no subjective adverse reactions, while 45 (54.2%) reported gastrointestinal disturbance, exhibiting stomach ache, acid regurgitation, nausea or diarrhea. Additionally, headache, dizziness and hepatic pain (or 'sting') each occurred in at least one case besides gastrointestinal disturbance. Evident alopecia was not reported and observed in the current study. Therapy with albendazole was ceased in 2 cases during follow-up, due to occurrence of serious dizziness and stomach ache. Transaminase levels were not monitored in the current study, due to the difficulty of doing liver function tests in the field.

## Discussion

Albendazole became available for treatment of human hydatid disease in 1983 [25], [26], and its effectiveness in cystic echinococcosis (CE) has been evaluated in a series of studies [27]–[30], from which approximately 30% of patients were cured, a further 30–50% were improved, and 20–40% remained unchanged following 3 months to 6 months treatment. However, 25% of albendazole (ABZ) treated patients relapsed in long-term follow-up studies [30]. This indicated the need for a prolonged treatment greater than the standard 3 to 6 months in some cases. In the present study, the effectiveness of albendazole was assessed in 49 Tibetan CE patients, detected after mass screening, using ultrasound during a 6 to 30 months regular treatment and based on the WHO consensus classification [12]. Therapy was continued until hydatid cyst morphology changed to a CE4 type (ie. a solid mass with or without partial calcifications), but further regular ultrasound follow-up was required. As a result, 32.7% of CE patients with regular albendazole therapy showed involution of cysts to a CE4 type (cured), 49.0% of CE cases exhibited the cyst degenerating into CE3 (improved), whereas 14.3% remained unchanged and 4.1% were observed to have enlarged cysts or have recurrence of daughter cysts. Our current study also indicated that the cure rate increased with a prolonged treatment course, but the patient group with >18 months albendazole course was more likely to exhibit a cyst that changed to a CE4 type (57.1%) compared to those (22.9%) ≤18 months. This observation suggested that longer-term albendazole treatment caused a greater positive therapeutic impact as has been observed in other CE case series [28], [31]. Though no recurrence of CE was detected in the cured patients during 6 to 24 months ultrasound follow up in the current study, further ultrasound examination is necessary for years in the future. In contrast spontaneous involution of CE without treatment was observed in 18.9% of CE patients in the current study, which was consistent with previous reports of natural degeneration of hydatid cysts, for example in 13.6% and 21% of cysts in CE patients from Kenya and Argentina, respectively [32], [33].

Many factors can influence the effectiveness of anti-hydatid treatment, for example previous studies demonstrated that benzimidazole treatment was more efficacious against smaller and younger cysts, and the type of cyst may also have an effect [1], [27]. Results from the current study indicated that patients with CE2 type cysts (ie. with daughter cysts) needed the longest treatment course (mean 26.4 months) before cure, whereas the curative duration was shortest (mean 9 months) in cases with CE3a or CE3b type cysts (ie. with evidence of detached membrane and/or degraded daughter cysts). In addition, our study also suggested that CE patients with large cysts (≥10 cm) needed a longer curative treatment period (mean 22.3 months), compared to patients with medium size (5–10 cm) cysts (mean 17.3 months) or those presenting with small (<5 cm) cysts (mean 6 months). Therefore, the putative duration of albendazole curative treatment may differ greatly in individual CE patients. Thus, it is strongly recommended that regular imaging monitoring, even in remote communities, be continued during the period of albendazole treatment to guide the appropriate medical treatment.

Antigen B (AgB), as a major component of *E. granulosus* hydatid cyst fluid, has been proved to have a high diagnostic value in the serological diagnosis of cystic echinococcosis in humans [34]–[37]. Our most recent serological study indicated that recombinant antigen B (rAgB) positive serum samples in ELISA were significantly lower in patients with CE1 type cysts, compared to other patients with CE2 or CE3 type cysts [38]. Similarly, in the current follow-up study only 46.2% (6/13) of patients with CE1 cyst showed positive antibody responses against rAgB before ABZ treatment. Interestingly, serum specific IgG antibody levels were observed to convert in six of the seven initially sero-negative CE1 patients following albendazole therapy. Concurrently, ultrasound scan revealed that hydatid cysts progressed from a CE1 type to a CE3a type in all these 6 CE patients with positive IgG antibody seroconversion. In the remaining one case (P7), specific serum antibody still remained negative by the end of follow-up, and ultrasound scan could not detect any improvement of the cyst. Subsequent questionnaire investigation showed that this patient failed to take albendazole as requested. This finding suggests that serum specific IgG antibody (against rAgB) was initially absent in a great proportion (53.8%) of CE1 patients, probably due to the typical intact cyst wall in this type of cyst which limits the release of antigenic cyst fluid into the circulation. Therefore, occurrence of serum specific IgG antibody in initially sero-negative CE1 hydatid patients after albendazole administration, not only conversely verified the clinical diagnosis, but importantly also acted as a positive indicator for the potential therapeutic effectiveness of antiparasitic drugs.

The present study on community detected Tibetan CE cases disclosed two broad profiles of specific IgG antibody dynamics in a majority of improved or cured CE patients during albendazole therapy. One profile was typified by specific antibody levels that were initially elevated then subsequently decreased, whereas the second antibody profile was characterized by progressive decline in serum IgG antibody levels. However, all cured CE patients still remained seropositive at the end of medical treatment. Therefore, assessment of serum rAgB-specific IgG antibody levels in this group of Tibetan CE patients could only provide limited information about the effectiveness of albendazole. Nevertheless, occurrence of post-chemotherapeutic IgG antibody seroconversion in CE1 patients appeared to be an important indicator for effectiveness of albendazole in such cases.

The most frequent adverse reactions associated with long-term albendazole treatment have been reported as gastrointestinal disturbances, reversible alopecia, elevation of liver transaminases, and pancytopenia [29], [39], [40]. In general, significant reversible abnormalities of liver function occur in about 20% of cases, reversible alopecia appears in about 5% of cases, while fatalities due to pancytopenia have so far been reported in only 3 cases of echinococcosis [29], [41]. In the current study, gastrointestinal upset was commonly reported by 54.2% of Tibetan CE patients taking oral ABZ therapy as out-patients, but was generally well tolerated by the majority. However, regular monitoring of parameters indicative of liver function were not carried out in the current community based study, as nomadic lifestyle and absence of medical facilities in this exceptional area limited the ability to use liver function testing. Importantly albendazole has proved to be relatively safe with normally only mild side-effects when they occurred [29], [42], [43].

In conclusion, this was the first attempt to assess the effectiveness of cyclic albendazole treatment for Tibetan CE patients treated as out-patients in their own communities. Though the sample size was small (n = 49), results from this study indicated that albendazole treatment showed beneficial efficacy in over 80% of CE patients, and the treatment course for a curative indication was strongly associated with hydatid cyst pathological type as well as the cyst size. In addition, the changes of specific serum IgG antibody levels against rAgB in CE patients provided a degree of limited but useful information about the effectiveness of albendazole during treatment. It should also be noted that nearly 19% of community diagnosed hepatic CE cases showed evidence of spontaneous cure. Further community based post-treatment follow-up studies of human CE needs to be continued in resource poor Tibetan areas of western China.

### Accession Number

The accession number in GenBank for recombinant antigen B applied in the current study is Z26336.
